# Successful Management of a Symptomatic Vallecular Cyst in an Adult: A Case Report

**DOI:** 10.7759/cureus.41829

**Published:** 2023-07-13

**Authors:** Salmah M Alharbi, Mohammed Alahmari, Mohammed Al Hamoud, Khalid Al Malwi, Hussain Abujamilah

**Affiliations:** 1 Otorhinolaryngology-Head and Neck Surgery Department, Aseer Central Hospital, Abha, SAU; 2 Anesthesia Department, Aseer Central Hospital, Abha, SAU

**Keywords:** dysphagia, dysphonia, vallecular cyst, symptomatic, microlaryngoscopy, electrocautery

## Abstract

In adults, vallecular cysts are usually asymptomatic but can present with respiratory and gastrointestinal manifestations. A 45-year-old man presented with a four-month progressive history of dysphonia, dysphagia to solid, snoring, choking, apnea, and aspiration. On examination, the patient was vitally stable with no remarkable local and physical examination. Flexible nasoendoscopy, computer tomography, and subsequent micro-laryngoscopy revealed a non-pulsating, non-congested 3 x 2 cm cyst obscuring vocal cord visualization. The cyst was removed completely by cold and hot techniques and was sent for biopsy. This case report presents the successful management of a symptomatic vallecular cyst through electrocautery.

## Introduction

A vallecular cyst is a rare anomaly in pediatrics and adults. It has different names, such as mucous retention cyst, pre-epiglottis cyst, epiglottis cyst, base-of-tongue cyst, and ductal cyst. Most cases in the current literature are presented as case reports that detail adult intubation challenges or occlusion of the newborn airway [1]. Vallecular cysts constitute about 5% of benign lesions of the larynx. Of the laryngeal cyst, vallecular cysts account for 10.5% to 20.1% of all laryngeal cysts [2,3]. In some instances, vallecular cysts typically cause only slight discomfort in the throat and are discovered through a normal conventional or endoscopic laryngeal check [4,5]. This study aimed to describe a symptomatic, large vallecular cyst that was successfully managed through resection by cold and hot techniques.

## Case presentation

**Figure 1:**
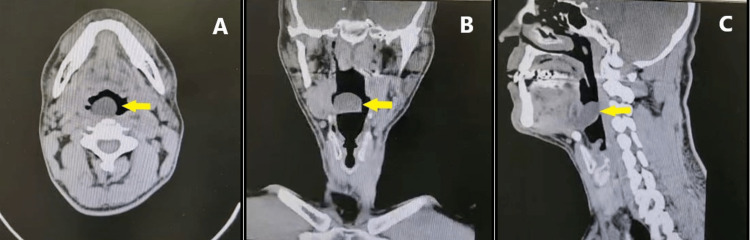
A preoperative computed tomography scan in the axial (a), coronal (b), and sagittal (c) planes of the head and neck showing a well-defined, 3 × 2 cm, hypodense cystic lesion on the vallecula (yellow arrow)

A 45-year-old soldier was referred from Mohail General Hospital with a history of progressive changing of his voice as well as dysphagia for solids for four months. Shortening of breath, aspiration, and frequent choking during sleep progressively developed over the next three months. Furthermore, he had witnessed apnea, daytime fatigue, and snoring. He was a smoker with a history of gastroesophageal reflux disease. There was no history of recent weight loss, night sweating, loss of appetite, fever, infection, surgery, hospitalization, previous same presentation, allergy, and no medical or family history. On examination, the patient was vitally stable with a pulse rate of 82 beats/minute, respiratory rate of 18 cycles/minute, systolic blood pressure of 132 mmHg, diastolic blood pressure of 79 mmHg, and oxygen saturation of 94% on room air. Intact cranial nerves and no lymphadenopathy were noted. The local and systemic examinations were unremarkable. Computed tomography on the head and neck revealed a well-defined 3 × 2 cm hypo-dense, cystic lesion on the vallecular, filling the airway with no other laryngeal abnormalities (Figure 1).

**Figure 2:**
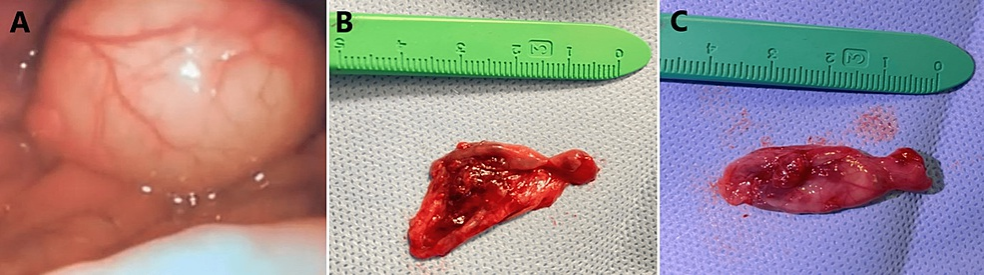
Intraoperative pictures with the micro laryngoscope showing a cystic lesion obscuring the vocal cords (A). The excised postoperative cystic lesion measured 3 × 2 cm (B and C)

Flexible nasoendoscoy revealed a non-pulsating, non-congested cystic mass originating from the vallecula, measuring 3 × 2 cm without any discharge. His airway was compromised, his vocal cords could not be seen, and signs of gastroesophageal reflux disease were detected (Figure 2).

**Figure 3:**
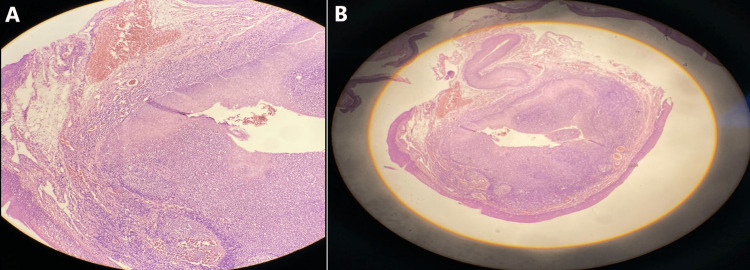
The histological appearance of a vallecular cyst that is lined with stratified squamous epithelium. Hematoxylin and eosin staining with magnification X100 (a) and X40 (b)

The patient was admitted for micro-laryngoscopy. Intubation was done by a bogie and 7.5 rigid laryngoscope tube. The vallecular mass was completely resected by cold and hot techniques and was sent for histopathological examination. Histopathological examination revealed a cystic lesion lined by stratified squamous epithelium with no malignancy observed (Figure 3).

Postoperative follow-up for one month revealed significant improvement without recurrence or aero-digestive symptoms.

## Discussion

Ductal and saccular cysts are types of laryngeal cysts. When the collecting ducts of the submucosal glands become obstructed and retain mucus, ductal cysts occur. Inflammation, irritation, or trauma may lead to the blocking of the duct [6]. Meanwhile, saccular cysts arise from the saccule that reaches the ventricle [7]. Ductal cysts are more common than saccular cysts [8]. The idea that the vallecular cyst is a dilated duct rather than a distended gland is supported by the fact that it is bordered with ductal cells (squamous or respiratory epithelium) rather than acinar cells [7].

In adulthood, vallecular cysts are more frequent but less hazardous. The majority of cysts affect men in the fifth decade of life [9]. The symptoms vary depending on the age of the patient as well as the site and size of the cysts. During the neonatal period, the cysts have been reported to cause noisy and labored breathing along with feeding difficulties. The other presentations in children are failure to thrive and/or laryngomalacia, which may further complicate the problem [10-12]. The cysts in adults are mostly asymptomatic and may be found as an incidental finding on X-ray or at the time of intubation for some surgical procedure. However, it may present with stridor, cough, dysphonia, feeling like a foreign body is in the throat, hoarseness, and dysphagia. These long-lasting mild symptoms from a non-infected vallecular cyst originate from pressure effects on nearby tissues. Secondary infection may develop in the epiglottitis, as an epiglottis abscess, or as more serious complications such as angioedema or abrupt death. It is advised that patients with a vallecular cyst along with acute epiglottitis with or without abscess formation seek an endoscopic follow-up of the vallecular region after being discharged to improve patient care [1]. In difficult circumstances, a vallecular cyst may be discovered when the patient poses a challenge for endotracheal intubation during rapid-sequence induction with general anesthesia [13]. Hence, even if the vallecular cyst is asymptomatic, it is recommended to remove the vallecular cyst by any technique [6].

Fiberoptic laryngoscopy can identify masses in the vallecula but cannot differentiate between a vallecular cyst, thyroglossal duct inner cyst, dermoid cyst, thyroid lingual tissue, lymphangioma, hemangioma, or teratoma [6]. Computed tomography scans can detect hidden vallecular cysts and can differentiate between vallecular cysts and mass lesions. Magnetic resonance imaging has the best diagnostic effectiveness in demonstrating smaller vallecular cysts and for antenatal diagnosis; however, it can be difficult in neonates and children [3]. Definitive diagnosis can easily be reached through an inspection of the tongue base with direct laryngoscopic examination using either flexible or rigid nasoendoscopy under general anesthesia [14]. In the current case study, nasoendoscopy was done prior to surgery to accurately evaluate and prepare for this case.

Intubation is usually a challenge. Fiberoptic intubation without a relaxant has been considered effective. However, it was difficult in some cases with a displaced larynx and large vallecular cyst. In some instances, rigid laryngoscopy and intubation can be effective. In neonates and infants, careful decompression of the cyst after local anesthesia could be successful but aspiration of a mucoid cyst is difficult. In most cases when all methods of intubation fail, tracheostomy is done [8]. In this case report, rigid laryngoscopy with a bogie and endotracheal intubation was successful.

For treating vallecular cysts, surgical procedures, including cyst aspiration, marsupialization, surgical debulking, and electrocautery or CO_2_ laser excision, can be used. Cyst aspiration is not advised as a final treatment because of the high likelihood of recurrence [13,15]. It is recommended to deroof the cyst under direct microlaryngoscopy (marsupialization) or through resection using an endoscopic CO_2 _laser. These techniques allow excellent depth perception, perfect surgical precision, and good control of postoperative edema and pain besides being suitable for all age groups [16-18]. In this case study, resection of the vallecular cyst through cold and hot electrocautery techniques had no adverse effects. It offered the benefit of being able to control bleeding from the surgical site during the removal of the lesion [19]. When the lesion was entirely removed, there was no evidence of vallecular cyst recurrence. The patient felt satisfied due to relief from the annoying complaint.

## Conclusions

A vallecular cyst is an uncommon lesion. Although it is usually asymptomatic, physicians should suspect the presence of a vallecular cyst when adult patients suffer from combined respiratory and gastrointestinal manifestations. Resection with the use of electrocautery is an effective management technique.
